# Comprehensive green growth indicators across countries and territories

**DOI:** 10.1038/s41597-023-02319-4

**Published:** 2023-06-24

**Authors:** Samuel Asumadu Sarkodie, Phebe Asantewaa Owusu, John Taden

**Affiliations:** 1 Nord University Business School (HHN), Post Box 1490, 8049 Bodø, Norway; 2 Pepperdine University, Malibu, California USA

**Keywords:** Sustainability, Environmental economics, Interdisciplinary studies

## Abstract

A sustainable transition to green growth is crucial for climate change adaptation and mitigation. However, the lack of clear and consistent definitions and common measures for green growth implies a disagreement on its determinants which hampers the ability to proffer valuable guidance to policymakers. We contribute to the global debate on green economic development by constructing green growth measures from 1990 to 2021 across 203 countries. The pillars of green growth are anchored on five dimensions namely natural resource base, socio-economic outcomes, environmental productivity, environmental-related policy responses, and quality of life. Contrary to the aggregated methods used in constructing indices in the extant literature, we employ a novel summary index technique with generalized least squares attributed-standardized-weighted index that controls for highly correlated variables and missing values. The constructed indicators can be used for both country-specific and global data modeling on green economic development useful for policy formulation.

## Background & Summary

Policies to promote green growth should be anchored on thorough knowledge and understanding of the concept. Likewise, tools to monitor green growth must have a reliable and comprehensive framework upon which progress can be recorded and compared across multiple entities. Yet, no two studies have a common measure or definition of green growth despite its widespread application in the scholarly and public policy discourse^1^. Not only does the lack of a common green growth measure stifle policymaking but also, hinders investments in its success^2^. The discourse on green growth is fairly new within international institutional development programs. As a multilateral agenda, green growth was first adopted in 2005 by 52 Asia-Pacific countries at Seoul’s 5^th^ Ministerial Conference on Environment and Development (MCED). The UN Economic and Social Commission for Asia and the Pacific (UNESCAP) then described the concept as a focus on sustained economic progress driven by environmental sustainability while improving low-carbon society and socially inclusive development (UNDESA, 2012, p. 35). The OECD in 2009 defined the term as achieving sustained economic development while reducing the negative environmental externalities including climate change, loss of biodiversity, and natural resource exploitation^3^. The organization also became the first to provide a cross-country ranking and comparative framework of green growth indicators for its industrialized economies in 2011^4^. The UN Environment Program (UNEP), under Towards Green Growth: Monitoring Progress program, also presented its first set of indicators in 2011. Per its definition, green economic growth improves well-being and social justice while reducing environmental risks and ecological footprint^5^. This infers that achieving green economic growth should prioritize green innovation, decarbonization, green trade, resource efficiency, and social inclusion^6^. Other international bodies such as the Global Green Growth Institute (GGGI), European Commission, and World Bank have all since proffered their definitions of the concept. In the wake of the discrepancies and unwieldy growth of the discourse surrounding green growth, the World Bank, UNIDO, OECD, and UNEP created the Green Growth Knowledge Platform (GGKP) in 2012. The GGKP is entrusted with the responsibility of collaboratively generating, managing, and sharing knowledge and data on green growth^7^. The GGGI produced the green growth index in 2019 aimed at preparing a measure that will enhance efforts to track the implementation of the Aichi Biodiversity Targets, Paris Accord, and SDGs^8^. At a country level, South Korea is widely recognized as the first to incorporate a comprehensive national strategy of green growth into its development plan in 2008^9^. Today, myriad countries across the globe have adopted various strategies to achieve green growth, even outside the frameworks of the SDGs and the Paris Climate Accord. Noticeably, developing countries such as Rwanda, Ethiopia, Vietnam, and Morocco have recently been commended for their track records on the incorporation of green growth into national development programs^10^.

In light of the growing adoption of green growth development into national development plans, researchers have recently focused on assessing performances and prospects of a green future across countries, but mostly relying on varied measures of the concept. For example, Houssini and Geng^10^ assessed Morocco’s green growth performance between 2000 and 2018 using a self-derived green growth measure developed with a data envelopment (D.E) analysis technique. The assessment proceeded to score Morocco positively on several variables, commending the government on its conscious effort to promote green growth. Wang and Shao^11^ developed the “Hybrid Global ML Index” to assess the effect of formal and informal national green growth performance among G20 economies. The study found that most countries in the G20 have achieved green growth benchmarks in the entire sample period, except from 2008 to 2009^11^. Several other studies used D.E analysis techniques but derived different green growth measures even when applied to the same units of analysis^12–16^. In related studies, Acosta, *et al*.^8^ assessed and ranked 115 countries in 2019 after using composite (C.O) analysis to develop a green growth index that strayed significantly from what its member partners such as the OECD and the UNEP historically relied upon. Their measure particularly paid attention to the inclusivity of green growth by being one of the first and only known two [with Kararach *et al*.^17^] to incorporate a gender dimension into the impacts of green growth policies. Though, using C.O analysis, other researchers^2^, however, arrived at measures that capture different sets of indicators for the same or different countries in their analyses.

**Fig. 1:**
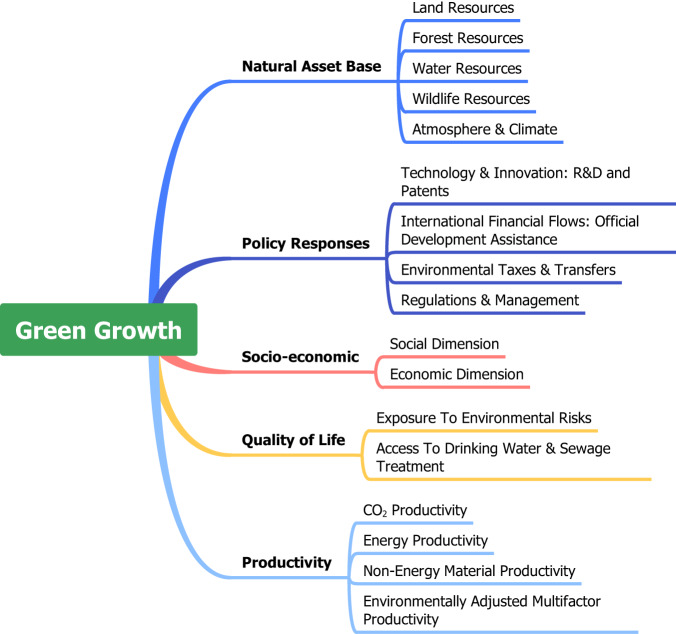
Dimensions of green growth. Data source: OECD (https://buff.ly/43cKbKU).

Patently, the lack of clear and consistent definitions and common measures for green growth imperils attempts to compare findings across multiple studies^1^, discourages investments in its success^2^, and forces the “comparison of apples to oranges” when analyzing the extant literature^1^. Likewise, the lack of a common understanding of the meaning of green growth and the utilization of myriad varying sets of indicators implies a lack of agreement on its determinants^2^, which hampers the ability to proffer valuable guidance to policymakers. In this study, we propose a new set of green growth indicators that is founded on a thorough assessment of a wider range of factors contributing to green growth. Theoretically, we portend that the dynamic shift from brown to green growth entails strategic multifaceted actions that are informed by economic endowments, political choices, socio-economic capabilities, and environmental outcomes. Accordingly, we define green growth as a sustained economic development approach decoupled from negative environmental consequences but thrives on eco-technological efficiency, reduces poverty, and increases social inclusion. Empirically, we construct green growth measures whose pillars are anchored on the five dimensions of (i) natural asset base, (ii) policy responses, (iii) socio-economic outcomes, (iv) quality of life, and (v) environmental productivity (see Fig. 1). Contrary to the aggregated methods used in constructing indices, we employ a novel summary index technique with generalized least squares attributed-standardized-weighted index that control for highly correlated variables and missing values.

## Methods

We analyze the frameworks and several indicators adopted by existing studies measuring green growth. For brevity, we analyze the indicators in light of our theoretical and empirical framework. Our empirical framework covers the dimensions of (i) natural asset base, (ii) policy responses, (iii) socio-economic outcomes, (iv) quality of life, and (v) environmental productivity (Fig. 1). To the best of our knowledge, these dimensions subsume the differing frameworks in the extant literature and, thus, allow us to review and analyze the individual indicators espoused in most studies. While not all studies in the extant literature are analyzed, Tables 1, 2 describe all known studies, their measurement frameworks, and other applications.

**Table 1:** Existing Measures of Green Growth (Panel A).

Authors	Framework/Structural Inputs	Indicators	Name	Purpose/Application	Method
Huang and Quibria^32^	(i) Productivity of environment and resources; (ii) Natural assets; (iii) Quality of life; (iv) Policy reactions; (v) socio-economic context	22	Index of Green Development	Determinants of G.G in BRICS and 42 OECD countries, [1990–2009]	C.O analysis
Kim, *et al*.^4^	(i) Production efficiency; (ii) Consumption efficiency; (iii) natural capital and quality of environment; (iv) quality of life; (v) Economic actors’ responses	12	G.G Status	Assessing G.G in 30 OECD countries	C.O analysis
Guo, *et al*.^33^	(i) Energy use per GDP (ii) CO_2_ per GDP	2	Regional G.G Performance	G.G performance, innovation and, environmental regulation in 30 provinces in China, [2011–12]	C.O analysis
Jha, *et al*.^34^	(i) Growth; (ii) Social equity; (iii) Environmental sustainability	28	Inclusive G.G Index	Applying IGGI in Asia and the OECD	C.O analysis
Kararach, *et al*.^17^	(i) Socioeconomic and growth features; (ii) Environmental and resource productivity; (iii) Natural asset monitoring; (iv) Gender; (v) Governance	48	African G.G Index	Reflections of AGGI in 22 African countries	C.O analysis
Lee and Chou^35^	(i) Environmental and resource productivity; (ii) Stock of natural resources; (iii) Quality of life; (iv) Green opportunities and policy reactions	20	G.G Index	Application of OECD indicators in Taiwan, [2002–2011]	C.O analysis
Acosta, *et al*.^8^	(i) Efficient and sustainable resource utilization; (ii) Protection of natural assets, (iii) Opportunities; (iv) Social inclusion	36	G.G Index	Assessing the G.G of 115 countries	C.O analysis
Šneiderienė, *et al*.^36^	(i) Economy; (ii) Society; and (iii) Environment	32	G.G Evaluation Index	Evaluation of the G.G of 27 EU countries	C.O analysis
Ates and Derinkuyu^2^	(i) Economic indicators; (ii) Environmental and resource productivity; (iii) Opportunities and policy reactions; (iv) Natural asset base	11	G.G Performance	Ranking OECD countries on G.G	C.O analysis
Baniya, *et al*.^18^	(i) Productivity of energy; (ii) Carbon productivity; (iii) Material productivity; (iv) Proportion of GDP from services; (v) Proportion of renewables; (vi) Forest coverage per total land*	6	Greening Growth	Development of G.G in Bangladesh and Nepal	C.O analysis
Gu, *et al*.^37^	(i) Growth; (ii) Social opportunity; (iii) Green production and green consumption; (iv) Protection of ecological environment	33	Inclusive G.G index	Economic policy uncertainty and G.G in 30 Chinese provinces, [2006–2016]	C.O analysis
Jadoon, *et al*.^38^	(i) Social equity; (ii) Performance of the economic; (iii) Performance of the environment	21	G.G Index	Impact of G.G on financial stability, [2010–15]	C.O analysis
Li, *et al*.^19^	(i) Prosperity, (ii) Inclusion, (iii) Utilization of resources, (iv) Sustainability of the environment	26	Inclusive G.G Indicator	Evaluation of IGG in the Asia-Pacific	C.O analysis
Liu, *et al*.^39^	(i) Economy, (ii) Opportunities, (iii) Green output and utilization, (iv) Environment	26	Inclusive G.G Index	Comparing IGG in the Yangtze River basin	C.O analysis
Wu and Zhou^40^	(i) Inclusiveness; (ii) Greenness; and (iii) Economic development	30	Inclusive G.G Index	Fiscal expenditure efficiency in China’s G.G, [2007–2018]	C.O analysis

Notes: *shows the authors directly used these indicators without a framework. G.G. is green growth; C.O. implies composite analysis; D.E. indicates data envelopment analysis; G.L.S denotes generalized-least squares approach. Source: Compilation by authors with adaptations from Leth^1^.

**Table 2:** Existing Measures of Green Growth (Panel B).

Authors	Framework/Structural Inputs	Indicators	Name	Purpose/Application	Method
Leth^1^	(i) Environmental, (ii) Social, (iii) Economic	27	G.G Score	A cross-country measure of G.G for 72 countries, [1990–2019]	C.O analysis
Zhang, *et al*.^41^	(i) Social dimension (ii) Environmental dimension	28	Inclusive G.G Index	IGGI’s impact of on China’s tourism, [2010–19]	C.O analysis
Zhu and Ye^12^	(i) Labor; (ii) Energy consumption; (iii) Capital	3	Inclusive G.G Index	Impact of FDI on IGG in Chinese provinces, [2000–2015]	D.E analysis
Wang and Shao^11^	(i) Labor; (ii) Capital; (iii) Energy	3	National G.G Level	Environmental regulations and G.G in the G20, [2001–2015]	D.E analysis
Cao, *et al*.^13^	(i) Capital; (ii) Labor; (iii) Innovation; (iv) Energy consumption	4	G.G Efficiency	Environmental regulation and G.G of manufacturing in China	D.E analysis
Chen, *et al*.^42^	(i) Capital; (ii) Labor; (iii) Land; (iv) Energy	4	Inclusive G.G Efficiency	Measurement of IGG in the Yangtze River Economic Belt Cities, [2003–2016]	D.E analysis
Qu, *et al*.^16^	(i) Capital; (ii) Energy; (iii) Labor	3	Manufacturing G.G	China’s manufacturing G.G and the global value chain	D.E analysis
Song, *et al*.^14^	(i) Labor; (ii) Capital	2	G.G Performance	Evaluation of green output in China	D.E analysis
Sun, *et al*.^15^	(i) Labor; (ii) Capital; (iii) Energy	3	Inclusive G.G	Measuring China’s regional IGG levels for 285 cities, [2003–2015]	D.E analysis
Houssini and Geng^10^	(i) Energy; (ii) Capital; (iii) Labor	3	G.G Efficiency	Evaluate performance of GG in Morocco, [2000–2018]	D.E analysis
Stoknes and Rockström^21^	Resource productivity	1	Genuine G.G	Nordic countries’ G.G evaluation, [2000–2015]	Single indicator as proxy
This study	The 152 variables are categorized into emissions productivity, energy productivity, non-energy material productivity, multifactor productivity, environmental risks, access to drinking water & sanitation, water resources, land resources, forest resources, wildlife resources, temperature, patents, Research and Development(R&D), Official Development Assistance (ODA), environmental taxes, environmental regulations, economic, and social context (See Supplementary Table 2 for detailed variables).	152	G.G Indicators	Comprehensive global G.G indicators for 203 countries and territories	Novel index construction using G.L.S approach

Notes: *shows the authors directly used these indicators without a framework. G.G. is green growth; C.O. implies composite analysis; D.E. indicates data envelopment analysis; G.L.S denotes generalized-least squares approach. Source: Compilation by authors with adaptations from Leth (2022).

### Natural asset base

Natural resources are central to the purpose of all green growth or sustainable development initiatives under the Aichi Biodiversity Targets, Paris Climate Accord, and SDGs^8^. The Green Growth Performance Measurement (GGPM) Program, for instance, developed in 2019 utilizes data from 115 countries and relies on 36 sampled indicators categorized under four dimensions. The dimensions include sustainable and efficient resource utilization, economic opportunities, natural capital protection, and social inclusion. Within the dimension of natural capital protection, for example, the authors discussed 12 indicators that reflect components such as the proportion of forests, biodiversity cover, and marine protected areas. However, the authors also incorporated measures such as the share of non-carbon agricultural emissions, mean annual air pollution, and the level of recreation and tourism in marine areas. By this calibration, the authors developed factors such as ecosystem, environmental quality, GHG reductions, and the cultural value of resources useful in measuring green growth under natural capital protection.

To design a novel tool to measure the complete impacts of green growth policies, Kim *et al*.^4^ synthesized a pool of 78 indicators in the extant literature down to what they describe as the 30 core and the 12 international indicators pertinent to assessing and comparing green growth across countries. Among the final 12 international indicators, two were used to measure the natural capital assets and environmental quality dimension which are: the inverse of domestic material consumption and the share of forest coverage per total land size. The authors’ omission of other non-forest resources in measuring natural capital is puzzling—as they offer no theoretical justification for the decision. However, the authors admit that by placing more weight on forest resources, the measure unfairly punishes countries with low forest cover such as Iceland. Baniya, *et al*.^18^ utilized six indicators to analyze green growth in Bangladesh and Nepal from 1985 to 2016—and to project its 2030 prospects in both countries. Natural capital was measured using the share of land with forest coverage. The authors argued that the six indicators chosen for analyses are the most frequently used in the extant literature from the OECD (2017) framework. However, similar to Kim *et al*.^4^, the omission of indicators capturing non-forest resources poses a challenge to the generalizability of the final measure of green growth.

Kararach, *et al*.^17^ developed the African Green Growth Index using data from 22 African countries. They featured 48 indicators representing five different dimensions including, socioeconomic context, productivity of resources and environment, natural asset base monitoring, gender, and governance. While natural assets include the usual measures of forests, land, agriculture, water, and aquatic resources, they also incorporated a disaster risk component. This measure tracks all disaster events from 1900 to 2014 and the population affected by disasters. Nonetheless, attempts to derive variables as far back as 1900 meant that the authors had to statistically impute a significant portion of the missing data, which admittedly leads to model uncertainties and affects the accuracy^17^. Inspired by the need to design a green growth index that accounts for the connection among society, economy, and nature, Li, *et al*.^19^ employed factor analysis to design an inclusive green growth indicator covering four unique dimensions, viz. social inclusion, economic security, resource use, and sustainability. However, the design failed to provide a stand-alone measure for natural capital. Instead, the authors created a resource use dimension that is measured by a derivation of energy consumption and variables of natural capital including arable land holdings, forest cover, land yield efficiency, and freshwater resources.

### Environmental productivity

The productivity dimension of green growth entails the efficient ways by which economic growth is decoupled from resource use. An existing study focused on both ecosystem services and the productive use of resources such as energy, water, and land^8^. The authors reinforced this dimension with eight indicators ranging from measures such as the share of primary energy provision to GDP to total material footprint per capita. Kim, *et al*.^4^ dedicated two of the five dimensions of their framework to measuring productivity. The first dimension labeled as environmental efficiency of production is proxied by two indicators namely GHG emissions per GDP as well as the share of GDP from services. The second dimension labeled as environmental efficiency of consumption is proxied by three indicators including energy utilization per GDP, the share of renewable energy consumed, and withdrawal of both surface and groundwater out of total available water. The authors argued that their framework aligns with that of the OECD and purposely adopts the growth-accounting approach to reach a broader context of global decision-making. However, this also implies that unique country characteristics of certain measures are glossed over for the sake of generalizability. Similarly, four of the six indicators that constitute Baniya, *et al*.’s^18^ framework have productivity connotations. These include the productivity of carbon, energy, materials, and the share of renewables. The authors, however, do not account for other crucial dimensions seen in other studies such as policy responses, quality of life, and social inclusion.

### Socio-economic dimension

New and sustainable economic opportunities are crucial to the success of green growth strategies. Thus, some authors incorporated socio-economic variables of green growth into their frameworks. For instance, Acosta, *et al*.^8^ employed a dimension termed green economic opportunities—that capture the share of economic opportunities that arise as investments shift from traditional activities to green sectors. They integrated this dimension with four indicators that capture environmental technology (i.e., number of patent publications), green employment, environmental goods exports, and net savings minus resource and pollution damages. On the other hand, Kararach, *et al*.^17^ introduced an entire dimension for gender, into which seven different indicators are fed. They argued that green growth must be assessed by its impact on societal inequalities. However, it is unclear why such indicators (i.e., female HIV prevalence, female labor force, female literacy, parliamentary seats, and ministerial positions held by women) were used to measure the gender dimension. Li, *et al*.^19^ argued that a green growth measure must quantitatively incorporate the themes of social equity, stability, and happiness. The authors provided an extensive set of indicators incorporated into the measure of social inclusion. However, unlike other studies that specifically addressed opportunities for women and minorities, Li, *et al*.^19^ does not specify what constitutes fair opportunities and what specific group of people development must consciously cater for.

### Quality of life

The quality-of-life dimension of green growth tracks individuals’ social well-being attributed to resources extracted for economic growth. The existing studies that incorporated aspects of quality of life conceptualize the measures with different but similar and overlapping terminologies. For example, Acosta, *et al*.^8^ combined variables that track economic and social well-being into a single dimension described as social inclusion. They argued that inequality and poverty are directly affected by the availability of resources and crucial basic services. Conversely, people rely heavily on the environment such as forests, wildlife resources, and among other natural assets for livelihoods. They postulated that the social performance of green growth should be measured because sustained economic growth requires reduced inequality^8^. Consequently, they classified concepts such as “access to basic services and resources, gender balance, social equity, and social protection” under the social inclusion dimension. The authors supported this dimension with 12 indicators ranging from “access to safe drinking water and sanitation” to “proportion of urban population living in slums”. While theoretically cogent, this dimension does not distinguish traditional economic conditions such as income levels from social conditions or quality of life such as health. Kim *et al*.^4^ measured the quality-of-life dimension with only one indicator, viz. the public transportation modal split. They justified the selection of this measure among other dimensions on a criterium of policy relevance, analytical soundness, and measurability.

### Policy responses

The policy response dimension of green growth is represented in fewer studies. Although the OECD framework incorporates a policy response dimension, the Green Growth Index developed by Acosta, *et al*.^8^ omits it. Among studies that justify the policy response dimension, Kim *et al*.^4^ measured the response with four indicators: environmental expenditure, environmental patents, green ODA per GDP, and green R&D per government budget. While Kararach, *et al*.^17^ does not incorporate a precise dimension for policy responses, they accentuated the distinct role of governance in their equation. Their “governance” dimension spells out four political indicators pertinent to measuring green growth. These indicators include “violence and/or terrorism, control of corruption, government effectiveness, political stability, rule of law, and regulatory quality”. Nonetheless, these variables primarily capture institutional quality and could have been complemented by other deliberate governmental efforts to promote green growth, such as eco-innovation R&D and environmental taxes.

### Analysis of the literature

No two studies in the extant literature have the same or directly comparable measures of green growth^1^. From our review, no two studies have a common understanding of what set of indicators constitutes green growth or dimensions of green growth. In other scenarios, indicators used as proxies for one dimension in a study—for example, resource productivity—might be adopted as proxies for an entirely different dimension (such as economic opportunities) in another study. Consequently, their differential weightings in different dimensions imply they feature at different levels of importance in different studies. In such scenarios, comparing findings in the literature using these different measures might be tantamount to comparing apples to oranges^1^.

A few existing studies introduced distinct dimensions in their frameworks for various reasons. For instance, Li, *et al*.^19^ utilized GDP as a measure of economic growth and termed as “economic prosperity”. They proceeded to introduce an economic prosperity dimension using indicators such as trade volumes, R&D expenditure, urbanization, and inflation in addition to GDP and GDP per capita. They argued that economic prosperity should be the primary criterion of green and inclusive growth, stating further that more focus must be placed on the growth, development, and stability potentials of the economy. Notably, these indicators were captured in different studies, even under different dimensions and for different conceptual purposes. Contrary to studies that employed C.O. analysis to measure green growth, a large share of the extant literature relies on D.E. analysis to derive the green growth index. Studies that used C.O. analysis adopted a range of diverse inputs. For instance, whereas Song, *et al*.^14^ used labor and capital as inputs in their model, Cao, *et al*.^13^ relied on capital, labor, technological innovation, and energy consumption. Tables 1, 2 provide more information on the variability of datasets (used as inputs) and techniques in the existing studies.

Beyond studies utilizing C.O analysis and D.E techniques to measure green growth, a few other studies insist on simplifying the discourse further by adopting a single indicator out of the larger literature as a proxy for green growth. Accordingly, some researchers measure green growth using single indicators such as environmental and resource productivity^20^, carbon productivity^21^, and environmentally-adjusted-multifactor productivity^22^. In earnest, while these measures simplify analyses of green growth, they risk revealing only a portion of the picture. For instance, the use of carbon emissions as a sole indicator for green growth does not reveal any information beyond the pollution levels of the set of tools applied to achieving a certain level of growth^23^. Consequently, the contributions of other factors such as economic opportunities, policy responses, and resource protection to green growth are untenably omitted.

### Index-making model

**Fig. 2:**
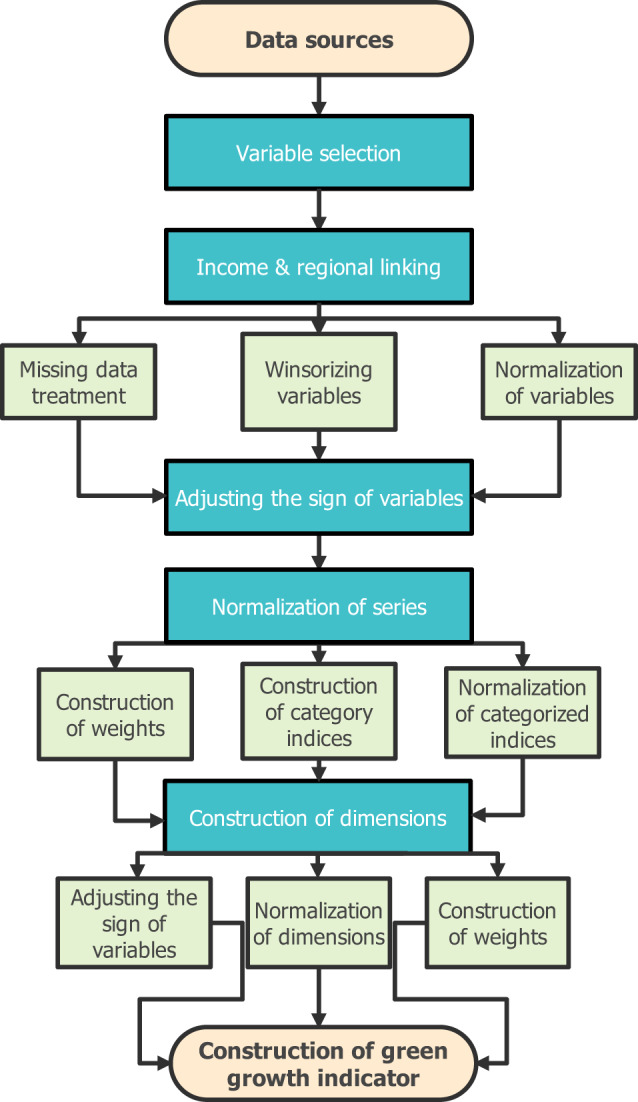
Green growth index-making methodological structure.

The schematic representation depicted in Fig. 2 shows the green growth index-making methodological structure used in this study. The steps include variable selection—used for categorization and classifying dimensions, income group & regional classifications, treatment of missing data, winsorization & normalization of data, adjusting the sign of variables where applicable, construction of weights, and construction of summary indices. While missing observations are often problematic in panel data, the estimation technique ignores missing outcomes (but also receives low weight) in creating new indices but utilizes all available data. Following the normalization procedure in developing the summary indicator, the technique further controls for missing data by fixing the values of the missing indicator to zero (0) — the mean of the reference group. This strategy further improves estimation efficiency by assigning lower weights to categories and dimensions with missing values^24^. The winsorizing technique entails generating new data by trimming 1^st^ and 99^th^ percentiles across countries and territories to treat extreme outliers^25^. Normalization $\left[{\rm{norm}}=\left({y}_{i}-{y}_{min}\right)/\left({y}_{max}-{y}_{min}\right)\right]$ involves the generation of new data by normalization of scores ranging between 0 and 1 using the minimum (*y*_*min*_) and maximum (*y*_*max*_) observations while accounting for periodic data frequency across countries and territories. The summary index approach used to construct the green growth indicators can be expressed as^24^:

$${\bar{s}}_{i,j}=\frac{1}{{W}_{i,j}}\sum _{k\in {{\mathbb{K}}}_{i,j}}{w}_{j,k}\frac{{y}_{i,j,k}-{\bar{y}}_{j,k}}{{\sigma }_{j,k}^{y}}$$

Where ${\bar{s}}_{i,j}$ is the constructed index (i.e., the outcomes can be categories, dimensions, and green growth indicators) with a weighted average *W*_*i,j*_. *w*_*j,k*_ denotes the weight of the normalized output $[({y}_{i,j,k}-{\bar{y}}_{j,k})/{{\sigma }}_{j,k}^{y}]$ from inverse covariance matrix ${\widehat{\Sigma }}_{j}^{-1}$, ${\widetilde{y}}_{i,j,k}$ is the inputs for individual *i*, indicators *k*, and country *j*. The normalized output is generated via effect sizes by dividing demeaned indicators $({y}_{i,j,k}-{\bar{y}}_{j,k})$ by the standard deviation ${\sigma }_{j,k}^{y}$ of the reference group, whereas ${{\mathbb{K}}}_{i,j}$ represents non-missing outcomes for indicators *k* and country *j*. The summary index combines several variables into categories and several categories into dimensions and subsequently develops the green growth indicators. This test involves: (1) adjusting the sign of variables to indicate a better result, thus, a positive direction of the series always has positive consequences on the environment. (2) normalizing indicators into similar scales (3) constructing weights (4) constructing indices using weighted average and (5) normalizing indices. Thus, the winsorized and normalized dataset was classified into categories, dimensions, and green growth indicators. Contrary to the aggregated methods used in constructing indices^26^, the novel summary index technique with generalized least squares (GLS) attributed-standardized-weighted index used in this study controls for highly correlated variables and missing values.

### Category indices of green growth

Our empirical assessment began with a panel-based descriptive statistical analysis of 152 raw data series spanning over 30 years. The descriptive statistical metrics led to the identification of several missing and extreme distributions requiring winsorizing. The winsorized and normalized dataset was classified into 18 category indices with a composition of the estimated weight of 152 variables. As the rule of the GLS summary index algorithm presented in Schwab, *et al*.^27^, highly-correlated variables were apportioned offsetting or small constructed weights whereas less-correlated or unique variables were apportioned higher constructed weights (Tables 3, 4). Positive weights signify a higher contribution to the summary index whereas negative weights signify factors that decrease the summary index. For example, demand-based CO_2_ emissions (CO2_DBEM) contribute the highest weight (1.447) to the summary emission index whereas production-based CO_2_ emissions (CO2_PBEM) show the highest weight (−0.865) that negatively enter the summary emission productivity index (Table 3). Energy consumption in other sectors (NRGC_OTH) and biomass (DMC_BIO) contribute the highest weights (0.350 and 0.278, respectively) to the energy productivity and non-energy material productivity indices whereas environmentally-adjusted multifactor productivity growth (EAMFP_EAMFPG) and relative advantage in environment-related technology (GPAT_DE_RTA) have the lowest weights (0.280 and 0.124, respectively) in the multifactor productivity and patents summary indices. Similarly, marine protected areas (PA_MARINE), allocable ODA to the environment sector (ODA_ENVSEC), and Petrol tax (FTAX_DIE_S) are assigned larger weights (0.520, 0.312, and 0.548, respectively) for environmental regulations, taxes & transfer, and official development assistance summary indices while purchasing power parity (PPP), environmentally-related R&D expenditure (ENVRD_GDP), and welfare costs of premature mortalities from exposure to PM_2.5_ (PM_SC) are assigned offsetting or small weights (−0.011, 0.079, and −1.859, respectively) for the economic dimension, environmental risks, and R&D summary indices. Besides, the population connected to public sewerage (ASEW_POP), permanent surface water (SW_PERMWAT), threatened vascular plant species (WLIFE_PL), and net migration (POP_NETMIGR) show the lowest weights (−0.144, −0.044, −0.025, and 0.070, respectively) assigned for the summary indices of access to drinking water & sanitation, water resources, wildlife resources, and social dimension (Table 4).

**Table 3:** Estimated weight of variables for category indices composition (Panel A).

Series	Weight	Series	Weight	Series	Weight
***Emissions***		***Taxes***		***Temperature***	
CO2_AIRTRACAP	−0.047	COAL_FFS	−0.005	TEMPCHANGE5180	1.000
CO2_AIRTRAGDP	0.065	CSE_ENET	0.004	***RND***	
CO2_DBEM	1.447	CSE_FFS	−0.005	ENVRD_GBAORD	0.083
CO2_DBEM00	0.011	CSE_TOTT	0.159	ENVRD_GDP	0.079
CO2_DBEMCAP	−0.008	ECR_PC120UP	0.008	ERD_GDP	0.365
CO2_DBPROD	0.069	ECR_PC30UP	0.115	FFRD_ERD	0.177
CO2_DBPROD_NNDI	−0.020	ECR_PC60UP	−0.014	RERD_ERD	0.296
CO2_INTPROD	0.121	ELEC_FFS	0.103	***Risks***	
CO2_PBEM	−0.865	ENVTAX_GDP	−0.002	O3_MOR	−0.805
CO2_PBEM00	0.126	ENVTAX_NRG	0.008	O3_SC	1.115
CO2_PBEMCAP	0.035	ENVTAX_TR	0.003	PB_MOR	0.337
CO2_PBPROD	0.067	ENVTAX_VEH	−0.003	PB_SC	0.259
***Energy***		EPRICE_IND	−0.029	PM_MOR	1.870
NRGC_AGR	0.063	EPRICE_RES	0.056	PM_PWM	−0.077
NRGC_IND	0.237	FFS_TTAX	−0.011	PM_SC	−1.859
NRGC_OTH	0.350	FIT_SOLAR	0.024	PM_SPEX10	0.044
NRGC_SER	0.108	FIT_WIND	−0.020	PM_SPEX35	0.100
NRGC_TRA	0.229	FPRICE_DIE	0.000	RN_MOR	−0.648
NRGS	0.002	FPRICE_PET	0.025	RN_SC	0.664
NRG_I00	0.006	FTAX_DIE	0.373	***Access***	
NRG_INT	−0.002	FTAX_DIE_S	0.548	ASEW_POP	−0.144
NRG_PROD	0.000	FTAX_PET	0.014	ASEW_PWT	0.277
RE_NRG	0.003	FTAX_PET_S	0.010	ASEW_SWT	0.391
RE_TPES	0.000	GSSE_FFS	−0.374	ASEW_TWT	0.494
RE_TPES_EBIOM	0.003	NATG_FFS	−0.011	SANI_SPOP	−0.017
		PET_FFS	−0.033		
		PSE_FFS	0.059		

Notes: The weights of Land & Forest resources using the generalized least-squares of index construction algorithm were excluded because the matrix has extremely large missing values. Hence, both indices were derived using the weighted average of normalized variables. The sum of the estimated weight of all variables in each category indices is equal to 1. Positive weights signify a higher contribution to the summary index whereas negative weights signify factors that decrease the summary index. The variable description is presented in Supplementary Table 2.

**Table 4:** Estimated weight of variables for category indices composition (Panel B).

Series	Weight	Series	Weight	Series	Weight
***Non-Energy***		***ODA***		***Water***	
DMC_BIO	0.278	ODA_BIO	−0.051	SW_NOTTOPERM	−0.026
DMC_MET	0.159	ODA_CCADP	0.104	SW_PERMTONOT	−0.077
DMC_MIN	0.278	ODA_CCMIT	−0.142	SW_PERMTOSEAS	0.010
DMC_PROD	0.007	ODA_DES	0.189	SW_PERMWAT	−0.044
MWAS_INC	0.082	ODA_ENV	0.028	SW_SEASTOPERM	0.235
MWAS_INT	0.129	ODA_ENVSEC	0.312	SW_SEASWAT	0.910
MWAS_LANDF	0.066	ODA_GNI	0.091	WATER_FW_TIRR	0.022
MWAS_RECO	−0.005	ODA_RE	0.267	WATER_FW_TR	−0.016
NBAL_HA	0.004	ODA_WATER	0.202	WATER_FWCAP	−0.015
PBAL_HA	0.002			WATER_STPC	0.001
***Multifactor***		***Economic***		***Wildlife***	
EAMFP_APAG	0.377	AGRGDP_PC	0.302	PEST_AGLAND	0.000
EAMFP_EAMFPG	0.280	DEF	0.013	WLIFE_BI	0.145
EAMFP_NKG	0.342	GDP_R	−0.005	WLIFE_MA	0.230
***Patents***		GDP_RCAP	0.300	WLIFE_PL	0.625
GPAT_DE_AI	0.324	INDGDP_PC	0.004	***Social***	
GPAT_DE_AT	0.266	LTAX_GDP	−0.005	POP	0.073
GPAT_DE_CAP	0.286	LTAX_TTAX	0.017	POP_FERTILITY	0.333
GPAT_DE_RTA	0.124	PPP	0.378	POP_LIFEEXP	0.404
***Regulations***		SRVGDP_PC	0.017	POP_NETMIGR	0.070
PA_MARINE	0.520	XR	−0.022	POPDEN	0.120
PA_TERRESTRIAL	0.480				

Notes: The weights of Land & Forest resources using the generalized least-squares of index construction algorithm were excluded because the matrix has extremely large missing values. Hence, both indices were derived using the weighted average of normalized variables. The sum of the estimated weight of all variables in each category indices is equal to 1. Positive weights signify a higher contribution to the summary index whereas negative weights signify factors that decrease the summary index. The variable description is presented in Supplementary Table 2.

### Constructing green growth Indicators

We constructed 10 green growth indicators (Models 1–10) using all five dimensions in Fig. 1 but with varying input characteristics for users to choose from. In Model 2, the characteristics of all five dimensions (i.e., environmental productivity, environmental quality, natural asset base, policy response, and socioeconomics) show better environmental performance. In Model 1, the characteristics of policy response, and socioeconomics dimensions show better environmental impacts but environmental productivity, environmental quality, and natural asset base worsen the environment. In Model 3, the characteristics of environmental quality, natural asset base, policy response, and socioeconomics improve the environment but environmental productivity declines environmental sustainability. In Model 4, the characteristics of environmental productivity, natural asset base, policy response, and socioeconomics improve the environment but the environmental quality dimension spurs environmental degradation. In Model 5, the characteristics of environmental productivity, environmental quality, policy response, and socioeconomics promote sustainable environment whereas natural asset base is detrimental to the environment. In Model 6, the characteristics of natural asset base, policy response, and socioeconomics reduces environmental degradation whereas environmental productivity and environmental quality increase pollution. In Model 7, environmental quality, policy response, and socioeconomics improve sustainability while environmental productivity and natural asset base hamper clean environment. In Model 8, environmental productivity, policy response, and socioeconomics decline environmental threats whereas environmental quality and natural asset base escalate environmental consequences. In Model 9, the characteristics of environmental productivity, environmental quality, natural asset base, policy response, and socioeconomics deteriorate the environment and thwart sustainable transition. Model 10 incorporates all conditions in Models 1–9 except that the average weights of all indicators was used for its construction.

**Fig. 3:**
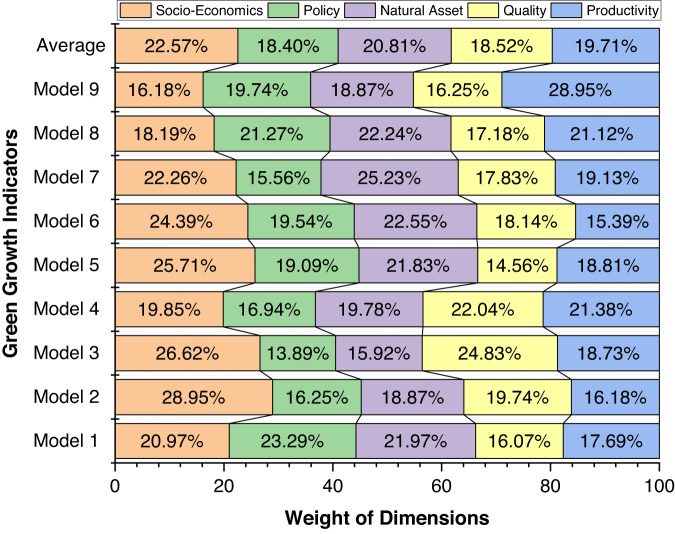
Weight of dimensions used to construct green growth indicators. Legend: Model 1- Comprise socioeconomics, policy response, natural asset base, environmental quality, and environmental productivity while altering the sign (i.e., flipping implies altering the sign to move in the opposite direction) of environmental productivity, environmental quality, and natural asset base. Model 2- Comprise socioeconomics, policy response, natural asset base, environmental quality, and environmental productivity with no flipping. Model 3- Comprise socioeconomics, policy response, natural asset base, environmental quality, and environmental productivity while altering the sign of environmental productivity. Model 4- Comprise socioeconomics, policy response, natural asset base, environmental quality, and environmental productivity while altering the sign of environmental quality. Model 5- Comprise socioeconomics, policy response, natural asset base, environmental quality, and environmental productivity while altering the sign of natural asset base. Model 6- Comprise socioeconomics, policy response, natural asset base, environmental quality, and environmental productivity while altering the sign of environmental productivity and quality. Model 7- Comprise socioeconomics, policy response, natural asset base, environmental quality, and environmental productivity while altering the sign of environmental productivity, and natural asset base. Model 8- Comprise socioeconomics, policy response, natural asset base, environmental quality, and environmental productivity while altering the sign of environmental quality, and natural asset base. Model 9- Comprise socioeconomics, policy response, natural asset base, environmental quality, and environmental productivity while altering the sign of socioeconomics, policy response, natural asset base, environmental quality, and environmental productivity. Model 10 (Average)- Comprise the average weight of socioeconomics, policy response, natural asset base, environmental quality, and environmental productivity in Models 1–9.

**Fig. 4:**
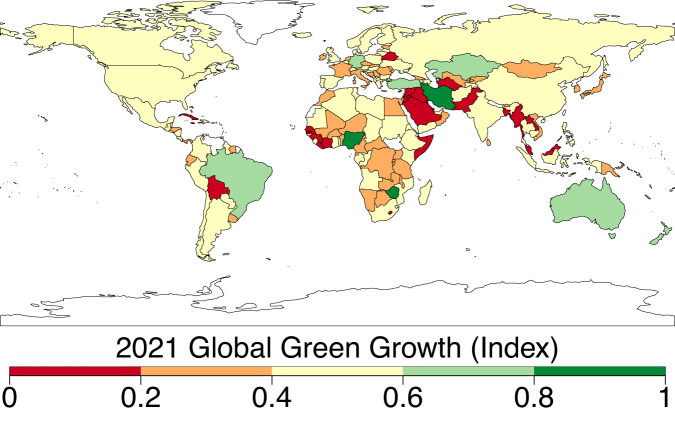
Global distribution of green growth (Index, Reference year: 2021, Model 2). Legend: *Score 0* implies low green growth performance whereas *Score 1* infers high green growth performance. The optimal green growth indicator (i.e., Model 2) captures 28.95% weight of socioeconomics dimension, 19.74% weight of environmental quality, 18.87% weight of natural asset base, 16.25% weight of policy response, and 16.18% weight of environmental productivity.

The negative characteristics of dimensions in Models 1–10 (used to construct the 10 green growth indicators) were determined using the flipping scenarios. The flipping scenarios used in calculating the ten green growth indicators are presented in Table 5. The parenthesis (…%) denotes the percentage weight of dimensions (in Fig. 3) from the model used to construct green growth indicators. Model 1 covers the role of policy response (23.29%), natural asset base (21.97%), socioeconomics (20.97%), environmental productivity (17.69%), and environmental quality (16.07%) while altering the sign (i.e., flipping implies altering the sign to move in the opposite direction) of environmental productivity, environmental quality, and natural asset base. Model 2 captures socioeconomics (28.95%), environmental quality (19.74%), natural asset base (18.87%), policy response (16.25%), and environmental productivity (16.18%) with no flipping option. Model 3 comprises socioeconomics (26.62%), environmental quality (24.83%), environmental productivity (18.73%), natural asset base (15.92%), and policy response (13.89%) while altering the sign of environmental productivity. Model 4 encompasses environmental quality (22.04%), environmental productivity (21.38%), socioeconomics (19.85%), natural asset base (19.78%), and policy response (16.94%) while altering the sign of environmental quality. Model 5 includes socioeconomics (25.71%), natural asset base (21.83%), policy response (19.09%), environmental productivity (18.81%), and environmental quality (14.56%) while altering the sign of natural asset base. Model 6 captures socioeconomics (24.39%), natural asset base (22.55%), policy response (19.54%), environmental quality (18.14%), and environmental productivity (15.39%) while altering the sign of environmental productivity, and environmental quality. Model 7 entails natural asset base (25.23%), socioeconomics (22.26%), environmental productivity (19.13%), environmental quality (17.83%), and policy response (15.56%) while altering the sign of environmental productivity, and natural asset base. Model 8 covers natural asset base (22.24%), policy response (21.27%), environmental productivity (21.12%), socioeconomics (18.19%), and environmental quality (17.18%) while altering the sign of environmental quality, and natural asset base. Model 9 includes environmental productivity (28.95%), policy response (19.74%), natural asset base (18.87%), environmental quality (16.25%), and socioeconomics (16.18%) while altering the sign of socioeconomics, policy response, natural asset base, environmental quality, and environmental productivity. Finally, the weight of dimensions used to construct green growth indicators reveals an average weight contribution (Model 10) of 22.57%, 20.81%, 19.71%, 18.52%, and 18.40% in the order of dimensions: socioeconomics > natural asset base > environmental productivity > environmental quality > policy response (Fig. 3). Figure 4 presents the optimal green growth index across countries for the period 2021. *Score 0* implies economies have low performance in transitioning toward green growth whereas *Score 1* infers countries have high performance toward green growth. The comparisons between countries for other green growth indicators are presented in Supplementary Fig. 1.

**Table 5:** Flipping scenarios used in calculating the ten green growth indicators.

Green Growth	*Socio-economics*	*Policy response*	*Natural asset base*	*Environmental quality*	*Environmental productivity*
Model 1	NA	NA	√	√	√
Model 2	NA	NA	NA	NA	NA
Model 3	NA	NA	NA	NA	√
Model 4	NA	NA	NA	√	NA
Model 5	NA	NA	√	NA	NA
Model 6	NA	NA	NA	√	√
Model 7	NA	NA	√	NA	√
Model 8	NA	NA	√	√	NA
Model 9	√	√	√	√	√
Average	NA	NA	NA	NA	NA

Notes: **√ **represents the reverse sign of dimensions in the variable list used in constructing the respective green growth indicator. NA implies no flip option of the specified dimension was incorporated in the estimation model. The green growth indicators namely Model 1, …, Model 9, and Model 10 (Average) are similar to the labeling such as GreenGrowth1, …., GreenGrowth9, and GreenGrowth10 available in the Figshare repository^28^.

## Data Records

We employed 152 variables (“OriginalData.xlsx”^28^) for 203 economies (see sampled countries with ISO3 code in Supplementary Table 1) based on the theoretical and empirical framework presented in Tables 1, 2 and Fig. 1 while integrating the sustainable development goals. We collected our global dataset spanning the period from 1990 to 2021 from OECD^29^ with an initial 16,184 observations. The data were sorted into 17 categories (see Fig. 2) namely emissions (12 variables), energy (12 variables), non-energy (10 variables), multifactor productivity (3 variables), environmental risks (11 variables), access (5 variables), water (10 variables), land (17 variables), forest (5 variables), wildlife (4 variables), temperature (1 variable), patents (4 variables), research & development [R&D] (5 variables), official development assistance [ODA] (9 variables), taxes (27 variables), regulations (2 variables), economic (10 variables), and social (5 variables) [see details of the 152 variables in Supplementary Table 2]. The 17 categories are subsequently classified into 5 dimensions, viz. natural assets (5 categories—water, land, forest, wildlife, and temperature), policy responses (5 categories—patents, R&D, ODA, taxes, and regulations), socio-economic (2 categories—social, and economic), quality of life (2 categories—environmental risks, and access), and environmental productivity (4 categories—emissions, energy, non-energy, and multifactor productivity). Finally, we use permutation and combination strategies detailed in subsequent sub-sections to construct 10 global indicators of green growth labeled as Model 1 (GreenGrowth1), Model 2 (GreenGrowth2), …, and Model 10 (GreenGrowth10). Table 6 shows the data description of constructed categories, dimensions, and green growth indicators—which are publicly available in the Figshare repository^28^.

**Table 6:** Data description of constructed variables.

Variable	Variable Name	Units	Source
***Categories***			
Emissions	Emissions Productivity	index (score: 0-1)	Authors
Energy	Energy Productivity	index (score: 0-1)	Authors
Non-Energy	Non-Energy Productivity	index (score: 0-1)	Authors
Multifactor	Multifactor Productivity	index (score: 0-1)	Authors
Risks	Environmental Risks	index (score: 0-1)	Authors
Access	Access to Water & Sanitation Quality	index (score: 0-1)	Authors
Water	Water Resources	index (score: 0-1)	Authors
Land	Land Resources	index (score: 0-1)	Authors
Forest	Forest Resources	index (score: 0-1)	Authors
Wildlife	Wildlife Resources	index (score: 0-1)	Authors
Temperature	Annual Surface Temperature since 1951–1980	index (score: 0-1)	Authors
Patents	Patents	index (score: 0-1)	Authors
RND	Research & Development	index (score: 0-1)	Authors
ODA	Official Development Assistance	index (score: 0-1)	Authors
Taxes	Environmental Taxes & Transfers	index (score: 0-1)	Authors
Regulations	Regulation & Management	index (score: 0-1)	Authors
Economic	Economic Context	index (score: 0-1)	Authors
Social	Social Context	index (score: 0-1)	Authors
***Dimensions***			
Socio-Economics	Socio-Economic Dimension	index (score: 0-1)	Authors
Policy	Policy Dimension	index (score: 0-1)	Authors
Natural Asset	Natural Asset Dimension	index (score: 0-1)	Authors
Quality	Environmental Quality Dimension	index (score: 0-1)	Authors
Productivity	Environmental Dimension	index (score: 0-1)	Authors
***Green Growth***			
Model 1	Green Growth 1	index (score: 0-1)	Authors
Model 2*	Green Growth 2 (Optimal indicator)	index (score: 0-1)	Authors
Model 3	Green Growth 3	index (score: 0-1)	Authors
Model 4	Green Growth 4	index (score: 0-1)	Authors
Model 5	Green Growth 5	index (score: 0-1)	Authors
Model 6	Green Growth 6	index (score: 0-1)	Authors
Model 7	Green Growth 7	index (score: 0-1)	Authors
Model 8	Green Growth 8	index (score: 0-1)	Authors
Model 9	Green Growth 9	index (score: 0-1)	Authors
Model 10 (Average)	Green Growth 10	index (score: 0-1)	Authors

Note: The constructed categories are used to construct dimensions whereas dimensions are used to construct the 10 green growth indicators via permutation and combination strategies in Table 5. * denotes the optimal green growth indicator that shows low (0) and (1) high green growth performance.

## Technical Validation

**Fig. 5:**
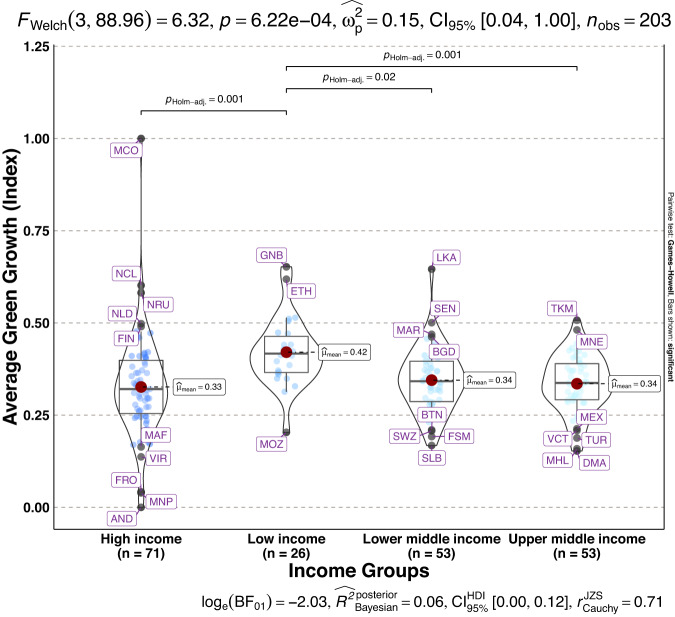
Statistical distribution of green growth indicator (Model 1) across income groups. Note: The country names with corresponding ISO3 codes are presented in Supplementary Table 1.

**Fig. 6:**
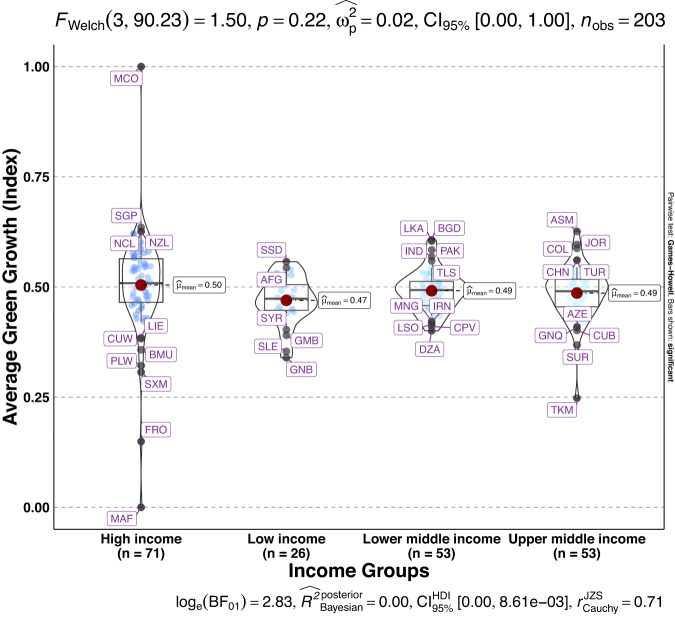
Statistical distribution of green growth indicator (Model 2) across income groups. Note: The country names with corresponding ISO3 codes are presented in Supplementary Table 1.

**Fig. 7:**
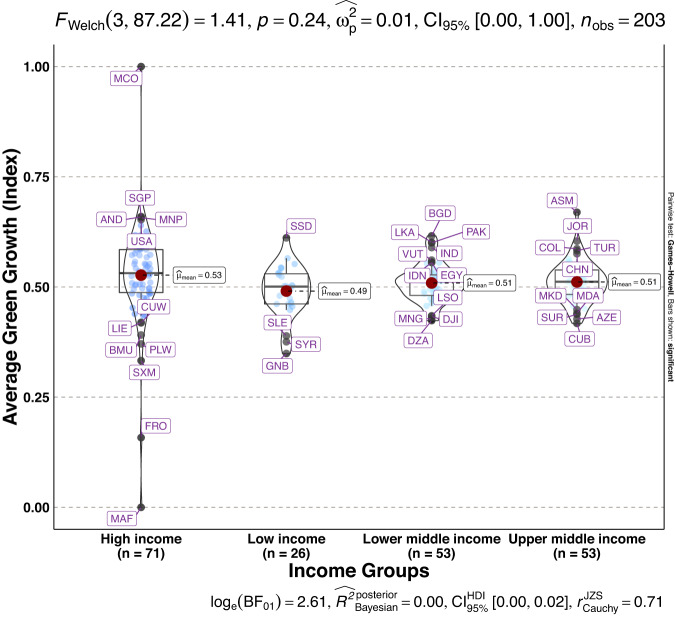
Statistical distribution of green growth indicator (Model 3) across income groups. Note: The country names with corresponding ISO3 codes are presented in Supplementary Table 1.

**Fig. 8:**
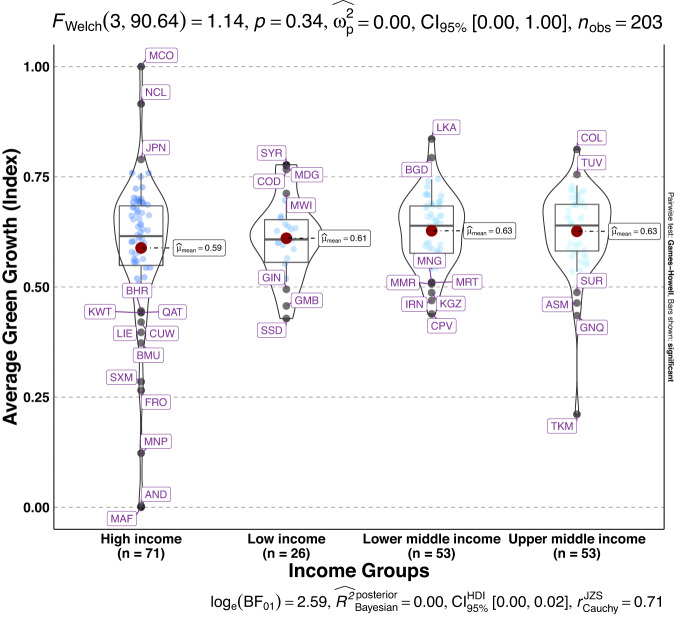
Statistical distribution of green growth indicator (Model 4) across income groups. Note: The country names with corresponding ISO3 codes are presented in Supplementary Table 1.

**Fig. 9:**
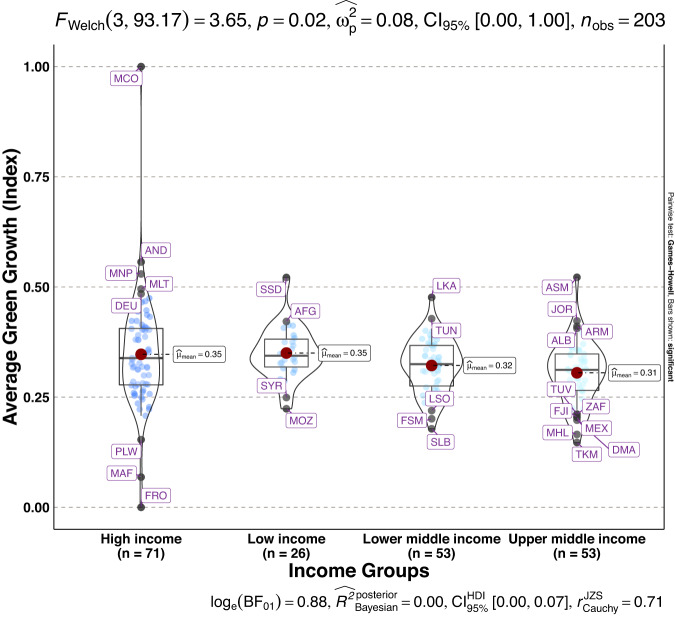
Statistical distribution of green growth indicator (Model 5) across income groups. Note: The country names with corresponding ISO3 codes are presented in Supplementary Table 1.

**Fig. 10:**
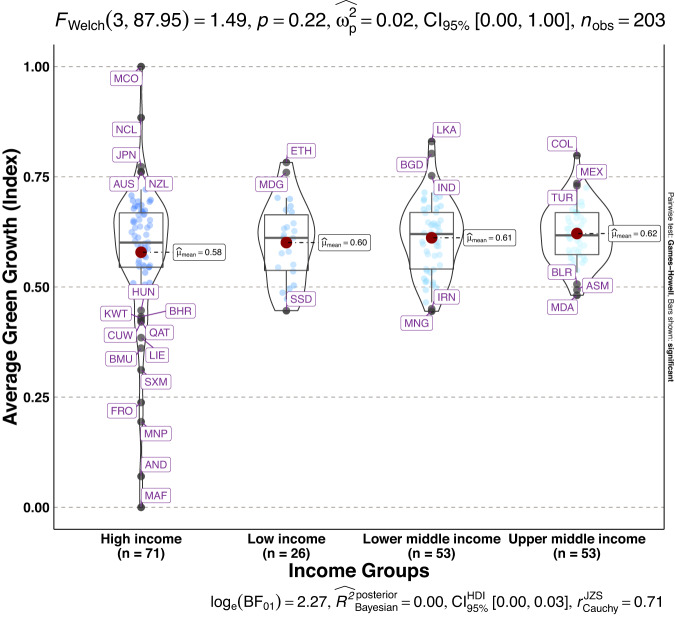
Statistical distribution of green growth indicator (Model 6) across income groups. Note: The country names with corresponding ISO3 codes are presented in Supplementary Table 1.

**Fig. 11:**
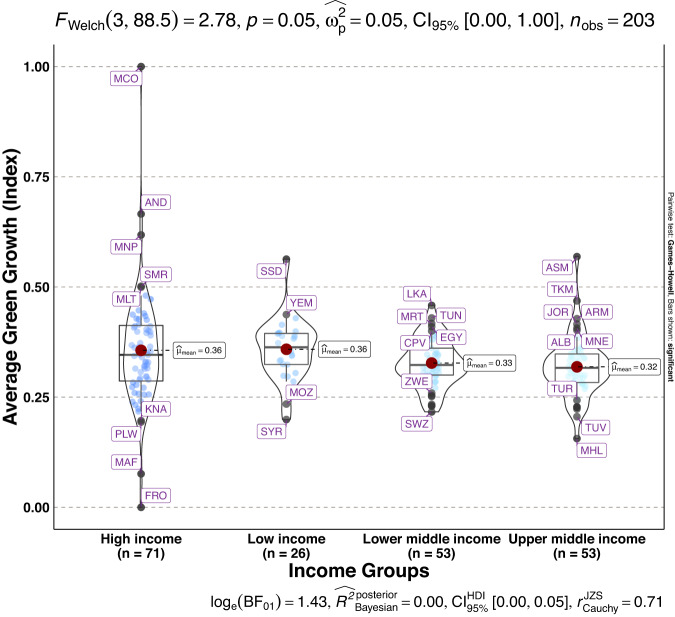
Statistical distribution of green growth indicator (Model 7) across income groups. Note: The country names with corresponding ISO3 codes are presented in Supplementary Table 1.

**Fig. 12:**
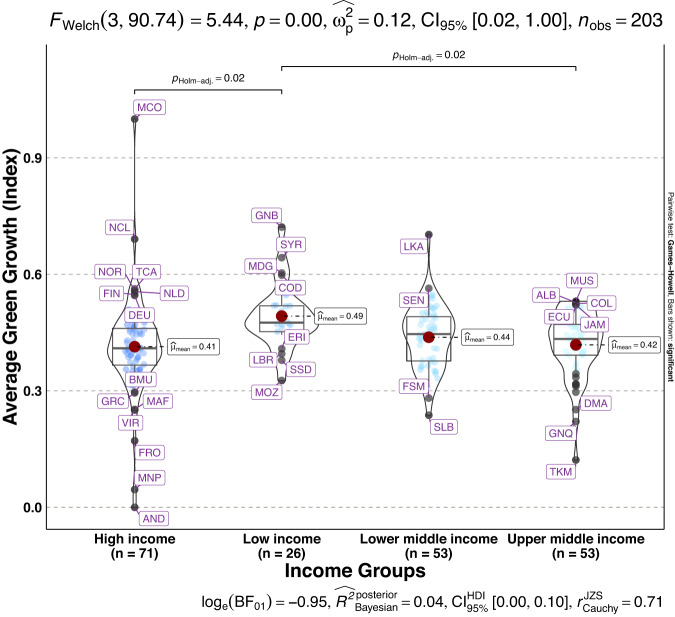
Statistical distribution of green growth indicator (Model 8) across income groups. Note: The country names with corresponding ISO3 codes are presented in Supplementary Table 1.

**Fig. 13:**
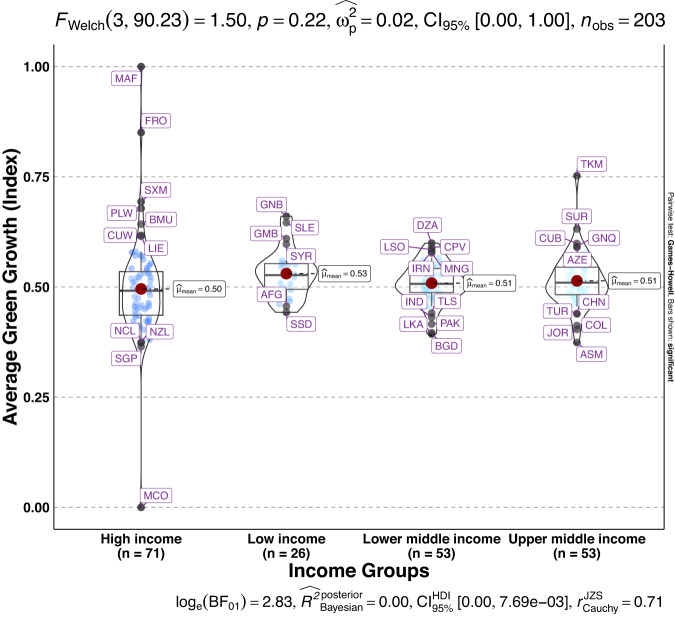
Statistical distribution of green growth indicator (Model 9) across income groups. Note: The country names with corresponding ISO3 codes are presented in Supplementary Table 1.

**Fig. 14:**
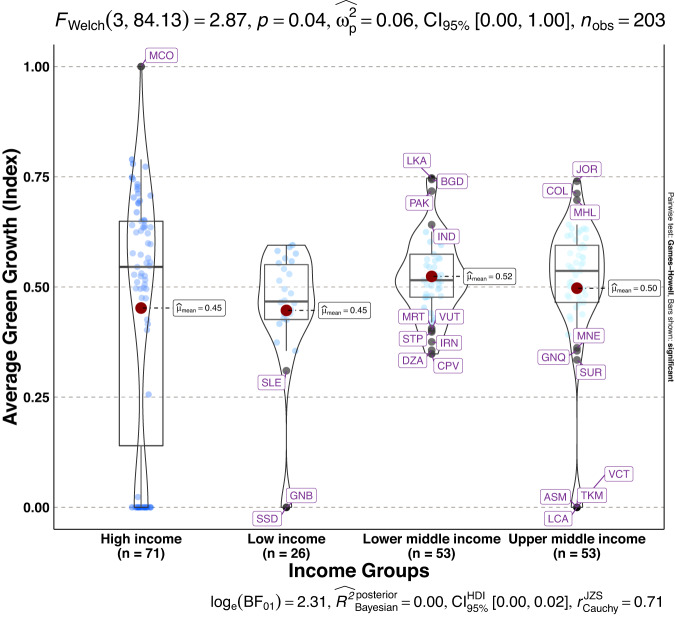
Statistical distribution of green growth indicator (Model 10) across income groups. Note: The country names with corresponding ISO3 codes are presented in Supplementary Table 1.

**Fig. 15:**
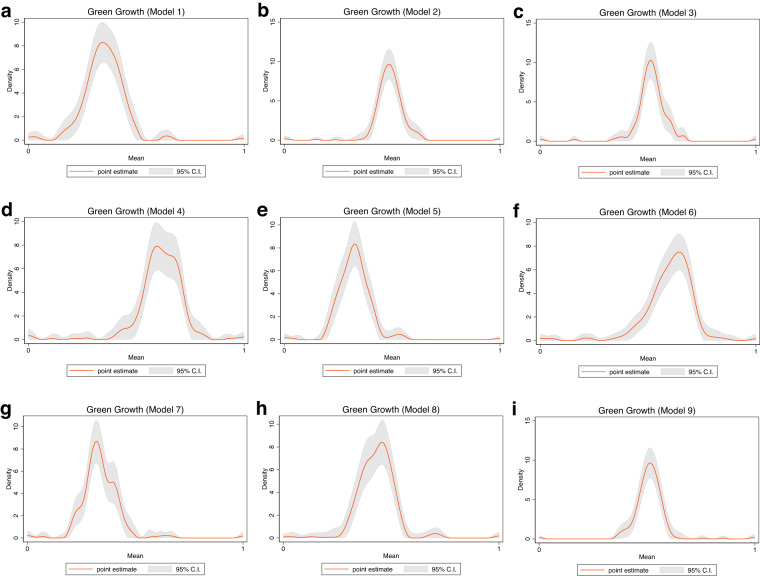
Panel heterogeneous effects of green growth indicators across economies (**a**) Model 1 (**b**) Model 2 (**c**) Model 3 (**d**) Model 4 (**e**) Model 5 (**f**) Model 6 (**g**) Model 7 (**h**) Model 8 (**i**) Model 9. Model 10 was ignored due to missing values with the function returning as an error.

To validate the quality of the dataset, we employed permutation and combination scenarios to construct 9 green growth indicators aside from the optimal indicator (labeled as Model 2). We further used statistical distribution^30^ to examine variations across income groups. The Games-Howell test shows that the pairwise distribution in Figs. 5–14 (excluding Figs. 5, 12) is not significantly different across groups, however, the mean of the optimal green growth (Fig. 6) indicator across income groups is in the descending order of high-income > upper-middle-income > lower-middle-income > low-income. This order is consistent with the environmental Kuznets curve (EKC) hypothesis^31^ which underscores improved environmental performance with rising income. Changes in the order across income groups in Figs. 5–14 (excluding Fig. 6) can be explained by the scenarios presented in Fig. 3. Yet, all constructed green growth indicators are heterogeneous across countries and territories (Fig. 15).

## Usage Notes

We developed 10 green growth indicators to examine the progress toward achieving a sustainable transition from a brown economy to a green economy. Each of the green growth indicators has underlying conditions and assumptions presented in Table 5 and Fig. 3. However, Model 2 (GreenGrowth2) is the most optimal green growth indicator showing economies with high scores have better performance and sustainable transition to green economic development. Evidence from Fig. 15 shows that future research that employs our datasets for panel data modeling should control for heterogeneous effects across economies. The caveat: there may be an underestimation of the standard errors of constructed dimensions and indicators because the GLS-derived weights are inclusively estimated parameters.

## Supplementary information

Supplementary Information

## Data Availability

No custom code was used to generate or process the data described in the manuscript—however, we used the “swindex” package in Stata [Stata/SE 17.0 for Mac (Intel 64-bit)] software to execute the steps detailed in the Methods section.
