# Supplementary material for: Comprehensive green growth indicators across countries and territories
Source: Sci Data. 2023 Jun 24;10:413. doi: 10.1038/s41597-023-02319-4 (PMC10290698; doi:10.1038/s41597-023-02319-4)
Supplement: Supplementary file 1 — Supplementary Information [file 41597_2023_2319_MOESM1_ESM.pdf]

Comprehensive green growth indicators across countries and territories

Samuel Asumadu Sarkodie[1]*, Phebe Asantewaa Owusu[2], John Taden[3]
[1, 2,] Nord University Business School (HHN). Post Box 1490, 8049 Bodø, Norway

[3,] Pepperdine University, Malibu California
*Email for correspondence: asumadusarkodiesamuel@yahoo.com

Supplementary Information

Table 1. Sampled countries with corresponding ISO3 code

Country	ISO3
American Samoa	ASM
Andorra	AND
Aruba	ABW
Bermuda	BMU
British Virgin Islands	VGB
Cayman Islands	CYM
Faeroe Islands	FRO
Liechtenstein	LIE
New Caledonia	NCL
Northern Mariana Islands	MNP
Puerto Rico	PRI
Saint Martin	MAF
San Marino	SMR
Sint Maarten	SXM
Turks and Caicos Islands	TCA
United States Virgin Islands	VIR
Nauru	NRU
Turkmenistan	TKM
Vanuatu	VUT
Equatorial Guinea	GNQ
Ethiopia	ETH
Palau	PLW
Fiji	FJI

Yemen	YEM
Antigua and Barbuda	ATG
Cabo Verde	CPV
Bahamas	BHS
Micronesia	FSM
Seychelles	SYC
Sao Tome and Principe	STP
Suriname	SUR
Montenegro	MNE
Gambia	GMB
Solomon Islands	SLB
Liberia	LBR
Samoa	WSM
Maldives	MDV
Tuvalu	TUV
Papua New Guinea	PNG
Niger	NER
Sierra Leone	SLE
South Sudan	SSD
Dominica	DMA
Mexico	MEX
Comoros	COM
Burundi	BDI
Kiribati	KIR
Tajikistan	TJK
Chad	TCD
South Africa	ZAF
Guinea	GIN
India	IND
Curacao	CUW
Turkey	TUR
Cambodia	KHM
Indonesia	IDN
Botswana	BWA
Lesotho	LSO
Luxembourg	LUX
Lao People's Democratic Republic	LAO

Libya	LBY
Myanmar	MMR
Peru	PER
Trinidad and Tobago	TTO
Guyana	GUY
Cuba	CUB
Rwanda	RWA
United States	USA
Armenia	ARM
Brazil	BRA
Spain	ESP
Bolivia	BOL
Bangladesh	BGD
Bhutan	BTN
Algeria	DZA
Belgium	BEL
Australia	AUS
Iran	IRN
Italy	ITA
Mauritania	MRT
Tonga	TON
Lebanon	LBN
Singapore	SGP
New Zealand	NZL
Belize	BLZ
Pakistan	PAK
Kazakhstan	KAZ
Central African Republic	CAF
Thailand	THA
Israel	ISR
Dominican Republic	DOM
Malawi	MWI
Ukraine	UKR
Mali	MLI
Finland	FIN
Argentina	ARG
Cyprus	CYP

Senegal	SEN
Greece	GRC
Kenya	KEN
China (People's Republic Of)	CHN
Burkina Faso	BFA
Mozambique	MOZ
Nigeria	NGA
Korea	KOR
Canada	CAN
Madagascar	MDG
Portugal	PRT
Bulgaria	BGR
Gabon	GAB
Bosnia and Herzegovina	BIH
Netherlands	NLD
Saudi Arabia	SAU
Zimbabwe	ZWE
Colombia	COL
Ghana	GHA
Eritrea	ERI
Sri Lanka	LKA
Egypt	EGY
Croatia	HRV
Qatar	QAT
Guatemala	GTM
United Kingdom	GBR
Barbados	BRB
United Arab Emirates	ARE
Bahrain	BHR
Uruguay	URY
Azerbaijan	AZE
Ireland	IRL
Afghanistan	AFG
Haiti	HTI
France	FRA
Russia	RUS
Kuwait	KWT

Mauritius	MUS
Romania	ROU
Malta	MLT
Uganda	UGA
Namibia	NAM
Morocco	MAR
Iceland	ISL
Zambia	ZMB
Austria	AUT
Tanzania	TZA
Slovenia	SVN
Poland	POL
Japan	JPN
Viet Nam	VNM
Chile	CHL
Albania	ALB
Czech Republic	CZE
Denmark	DNK
Germany	DEU
Switzerland	CHE
Oman	OMN
Sweden	SWE
El Salvador	SLV
Malaysia	MYS
Ecuador	ECU
North Macedonia	MKD
Tunisia	TUN
Iraq	IRQ
Jordan	JOR
Costa Rica	CRI
Hungary	HUN
Serbia	SRB
Kyrgyzstan	KGZ
Belarus	BLR
Nicaragua	NIC
Estonia	EST
Angola	AGO

Honduras	HND
Lithuania	LTU
Brunei Darussalam	BRN
Côte d'IVOIRE	CIV
Philippines	PHL
Slovak Republic	SVK
Paraguay	PRY
Syrian Arab Republic	SYR
Togo	TGO
Moldova	MDA
Congo	COG
Georgia	GEO
Cameroon	CMR
Panama	PAN
Latvia	LVA
Eswatini	SWZ
Mongolia	MNG
Uzbekistan	UZB
Benin	BEN
Jamaica	JAM
Sudan	SDN
Democratic Republic of the Congo	COD
Norway	NOR
Marshall Islands	MHL
Guinea-Bissau	GNB
Djibouti	DJI
Timor-Leste	TLS
Saint Vincent and the Grenadines	VCT
Monaco	MCO
Somalia	SOM
Saint Kitts and Nevis	KNA
Saint Lucia	LCA

Table 2. Sampled variables for developing economic growth indicators

Categories	Variable	Variable Name	Unit	Dimensions
**Emissions**	CO2_AIRTRACAP	CO_2 Emissions From Air Transport Per Capita	tonnes	**Environmental and resource productivity**
	CO2_AIRTRAGDP	CO_2 Emissions From Air Transport Per Unit of GDP	kilogram (kg), 2015	
	CO2_DBEM	Demand-Based CO_2 Emissions	tonnes, Millions	
	CO2_DBEM00	Demand-Based CO_2 Emissions, Index 2000=100	Index, 2000=100	
	CO2_DBEMCAP	Demand-Based CO_2 Intensity, Energy-Related CO_2 Per Capita	tonnes	
	CO2_DBPROD	Demand-Based CO_2 Productivity, GDP Per Unit of Energy-Related CO_2 Emissions	US$ per kg, 2015	
	CO2_DBPROD_NNDI	Demand-Based CO_2 Productivity, Disposable Income Per Unit of Energy-Related CO_2 Emissions	US$ per kg, 2015	
	CO2_INTPROD	CO_2 Intensity of GDP, CO_2 Emissions Per Unit of GDP	NA	
	CO2_PBEM	Production-Based CO_2 Emissions	tonnes, Millions	
	CO2_PBEM00	Production-Based CO_2 Emissions, Index 2000=100	Index, 2000=100	
	CO2_PBEMCAP	Production-Based CO_2 Intensity, Energy-Related CO_2 Per Capita	tonnes	
	CO2_PBPROD	Production-Based CO_2 Productivity, GDP Per Unit of Energy-Related CO_2 Emissions	US$ per kg, 2015	
**Energy**	NRG_I00	Total Primary Energy Supply, Index 2000=100	Index, 2000=100	
	NRG_INT	Energy Intensity, Tpes Per Capita	tonnes of oil equivalent (toe)	
	NRG_PROD	Energy Productivity, GDP Per Unit of Tpes	US$, 2015	
	NRGC_AGR	Energy Consumption in Agriculture	% Total Energy Consumption	
	NRGC_IND	Energy Consumption in Industry	% Total Energy Consumption	
	NRGC_OTH	Energy Consumption in Other Sectors	% Total Energy Consumption	
	NRGC_SER	Energy Consumption in Services	% Total Energy Consumption	

	NRGC_TRA	Energy Consumption in Transport	% Total Energy Consumption	
	NRGS	Total Primary Energy Supply	toe, Millions	
	RE_NRG	Renewable Electricity	% Total Electricity Generation	
	RE_TPES	Renewable Energy Supply	% Total Energy Supply	
	RE_TPES_EBIOM	Renewable Energy Supply (Excluding Solid Biofuels)	% Total Energy Supply	
**Non-Energy**	DMC_BIO	Biomass	% of DMC	
	DMC_MET	Metals	% of DMC	
	DMC_MIN	Non-Metallic Minerals	% of DMC	
	DMC_PROD	Non-Energy Material Productivity, GDP Per Unit of DMC	US$ per kg, 2015	
	MWAS_INC	Municipal Waste Incinerated	% Treated Waste	
	MWAS_INT	Municipal Waste Generated	kg per capita	
	MWAS_LANDF	Municipal Waste Disposed to Landfills	% Treated Waste	
	MWAS_RECO	Municipal Waste Recycled or Composted	% Treated Waste	
	NBAL_HA	Nitrogen Balance Per Hectare	kg	
	PBAL_HA	Phosphorus Balance Per Hectare	kg	
**Multifactor**	EAMFP_APAG	Adjustment for Pollution Abatement	%	
	EAMFP_EAMFPG	Environmentally Adjusted Multifactor Productivity Growth	%	
	EAMFP_NKG	Contribution of Natural Capital	%	
**Risks**	O3_MOR	Mortality From Exposure to Ambient Ozone	Per 1,000,000 inhabitants	**Environmental context of quality of life**
	O3_SC	Welfare Costs of Premature Deaths From Exposure to Ambient Ozone, GDP Equivalent	%	
	PB_MOR	Mortality From Exposure to Lead	Per 1,000,000 inhabitants	
	PB_SC	Welfare Costs of Premature Deaths From Exposure to Lead, GDP Equivalent	%	
	PM_MOR	Mortality From Exposure to Ambient $PM_{2.5}$	Per 1,000,000 inhabitants	
	PM_PWM	Mean Population Exposure to $PM_{2.5}$	μ/m^3	

	PM_SC	Welfare Costs of Premature Mortalities From Exposure to Ambient $PM_{2.5}$, GDP Equivalent	%	
	PM_SPEX10	Percentage of Population Exposed to More Than 10 $Micrograms/M^3$	%	
	PM_SPEX35	Percentage of Population Exposed to More Than 35 $Micrograms/M^3$	%	
	RN_MOR	Mortality From Exposure to Residential Radon	Per 1,000,000 inhabitants	
	RN_SC	Welfare Costs of Premature Mortalities From Exposure to Residential Radon, GDP Equivalent	%	
Access	ASEW_POP	Population Connected to Public Sewerage	% Total Population	
	ASEW_PWT	Population Connected to Sewerage With Primary Treatment	% Total Population	
	ASEW_SWT	Population Connected to Sewerage With Secondary Treatment	% Total Population	
	ASEW_TWT	Population Connected to Sewerage With Tertiary Treatment	% Total Population	
	SANI_SPOP	Population With Access to Improved Sanitation	% Total Population	
Water	SW_NOTTOPERM	Conversion of Not-Water to Permanent Water Surface	% Permanent Water, Since 1984	**Natural asset base**
	SW_PERMTONOT	Conversion of Permanent Water to Not-Water Surface	% Permanent Water, Since 1984	
	SW_PERMTOSEAS	Conversion of Permanent to Seasonal Water Surface	% Permanent Water, Since 1984	
	SW_PERMWAT	Permanent Surface Water	% Total Surface	
	SW_SEASTOPERM	Conversion of Seasonal to Permanent Water Surface	% Permanent Water, Since 1984	
	SW_SEASWAT	Seasonal Surface Water	% Total Surface	
	WATER_FW_TIRR	Water Stress, Total Freshwater Abstraction	% Total Internal Renewable Resources	
	WATER_FW_TR	Water Stress, Total Freshwater Abstraction	% Total Available Renewable Resources	

	WATER_FWCAP	Total Freshwater Abstraction Per Capita	Cubic metres per capita
	WATER_STPC	Total Renewable Freshwater Per Capita	Cubic metres per capita
Land	BA_BUILTA	Built Up Area	% Total Land
	BA_BUILTA_CAP	Built Up Area Per Capita	m^2
	BA_NEWBUILTA00	New Built Up Area	% Since 2000
	BA_NEWBUILTA90	New Built Up Area	% Since 1990
	IRRIG_AGLAND	Irrigated Land	% Agricultural Land
	LC_BAR	Bare Land	% Total
	LC_CRO	Cropland	% Total
	LC_NAT	Natural and Semi-Natural Vegetated Land	% Total
	LC_URB	Artificial Surfaces	% Total
	LC_WAT	Water	% Total
	LCC_CROPTOARTIF	Conversion From Cropland to Artificial Surfaces	% Since 1992
	LCC_NATGAIN04	Gain of Natural and Semi-Natural Vegetated Land	% Since 2004
	LCC_NATGAIN92	Gain of Natural and Semi-Natural Vegetated Land	% Since 1992
	LCC_NATLOSS04	Loss of Natural and Semi-Natural Vegetated Land	% Since 2004
	LCC_NATLOSS92	Loss of Natural and Semi-Natural Vegetated Land	% Since 1992
	LCC_NATTOARTIF	Conversion From Natural and Semi-Natural Land to Artificial Surfaces	% Since 1992
	LCC_NATTOCROP	Conversion From Natural and Semi-Natural Land to Cropland	% Since 1992
Forest	FOR_CER	Forests Under Sustainable Management Certification Fsc	% Total Forest Area
	FOR_IF_EXTENT	Intact Forest Landscape	km^2
	FOR_IF_PCLOSS2000	Intact Forest Landscape Loss	% Since 2000
	FOR_LTMGMT	Forests With Long-Term Management Plans	% Total Forest Area
	FOR_NAT	Naturally Regenerating Forests	% Total Forest Area
Wildlife	PEST_AGLAND	Sales of Pesticides Per Unit of Agricultural Land	kg
	WLIFE_BI	Threatened Bird Species	% Total Known Species
	WLIFE_MA	Threatened Mammal Species	% Total Known Species

	WLIFE_PL	Threatened Vascular Plant Species	% Total Known Species	
**Temperature**	TEMPCHANGE5180	Annual Surface Temperature, Change Since 1951-1980	Number	
**Patents**	GPAT_DE_AI	Development of Environment-Related Technologies	% Inventions Worldwide	**Economic opportunities and policy responses**
	GPAT_DE_AT	Development of Environment-Related Technologies	% All Technologies	
	GPAT_DE_CAP	Development of Environment-Related Technologies, Inventions Per Capita	Number	
	GPAT_DE_RTA	Relative Advantage in Environment-Related Technology	ratio	
**RND**	ENVRD_GBAORD	Environmentally Related Government R&d Budget	% Total Government R&d	
	ENVRD_GDP	Environmentally Related R&d Expenditure	% GDP	
	ERD_GDP	Energy Public Rd&d Budget	% GDP	
	FFRD_ERD	Fossil Fuel Public Rd&d Budget (Excluding Ccs)	% Total Energy Public Rd&d	
	RERD_ERD	Renewable Energy Public Rd&d Budget	% Total Energy Public Rd&d	
**ODA**	ODA_BIO	Oda - All Sectors - Biodiversity	% Total Oda	
	ODA_CCADP	Oda - All Sectors - Climate Change Adaptation	% Total Oda	
	ODA_CCMIT	Oda - All Sectors - Climate Change Mitigation	% Total Oda	
	ODA_DES	Oda - All Sectors - Desertification	% Total Oda	
	ODA_ENV	Environmentally Related Oda	% Total Oda	
	ODA_ENVSEC	Oda - Environment Sector	% Total Allocable Oda	
	ODA_GNI	Net Oda Provided	% Gni	
	ODA_RE	Oda - Renewable Energy Sector	% Total Allocable Oda	
	ODA_WATER	Oda - Water Supply and Sanitation Sector	% Total Allocable Oda	
**Taxes**	COAL_FFS	Coal Support	% Total Fossil Fuel Support	

CSE_ENET	Fossil Fuel Consumer Support	% Energy Related Tax Revenue
CSE_FFS	Fossil Fuel Consumer Support	% Total Fossil Fuel Support
CSE_TOTT	Fossil Fuel Consumer Support	% Total Tax Revenue
ECR_PC120UP	Emissions Priced Above Eur 120 Per Tonne of CO_2	% Total Emissions
ECR_PC30UP	Emissions Priced Above Eur 30 Per Tonne of CO_2	% Total Emissions
ECR_PC60UP	Emissions Priced Above Eur 60 Per Tonne of CO_2	% Total Emissions
ELEC_FFS	Electricity Support	% Total Fossil Fuel Support
ENVTAX_GDP	Environmentally Related Taxes	% GDP
ENVTAX_NRG	Energy Related Tax Revenue	% Total Environmental Tax Revenue
ENVTAX_TR	Environmentally Related Taxes	% Total Tax Revenue
ENVTAX_VEH	Road Transport-Related Tax Revenue	% Total Environmental Tax Revenue
EPRICE_IND	Industry Electricity Price	US$ Per kWh
EPRICE_RES	Residential Electricity Price	US$ Per kWh
FFS_TTAX	Total Fossil Fuel Support	% of Total Tax Revenue
FIT_SOLAR	Mean Feed-In Tariff for Solar PV Electricity Generation	US$
FIT_WIND	Mean Feed-In Tariff for Wind Electricity Generation	US$
FPRICE_DIE	Diesel End-User Price	US$ per litre, 2015
FPRICE_PET	Petrol End-User Price	US$ per litre, 2015
FTAX_DIE	Diesel Tax	US$ per litre, 2015
FTAX_DIE_S	Diesel tax	% end-user price
FTAX_PET	Petrol Tax	US$ per litre, 2015
FTAX_PET_S	Petrol tax	% end-user price
GSSE_FFS	Fossil Fuel General Services Support	% Total Fossil Fuel Support
NATG_FFS	Gas Support	% Total Fossil Fuel Support

	PET_FFS	Petroleum Support	% Total Fossil Fuel Support	
	PSE_FFS	Fossil Fuel Producer Support	% Total Fossil Fuel Support	
**Regulations**	PA_MARINE	Marine Protected Area	% Total Exclusive Economic Zone	
	PA_TERRESTRIAL	Terrestrial Protected Area	% Land Area	
**Economic**	AGRGDP_PC	Value Added in Agriculture	% of Total Value Added	**Socio-economic dimension**
	DEF	GDP Deflator	NA	
	GDP_R	Real GDP	NA	
	GDP_RCAP	Real GDP Per Capita	US$, 2015	
	INDGDP_PC	Value Added in Industry	% of Total Value Added	
	LTAX_GDP	Labour Tax Revenue	% GDP	
	LTAX_TTAX	Labour Tax Revenue	% Total Tax Revenue	
	PPP	Purchasing Power Parity	NA	
	SRVGDP_PC	Value Added in Services	% of Total Value Added	
	XR	Nominal Exchange Rate	NA	
**Social**	POP	Population	Per 1,000 inhabitants	
	POP_FERTILITY	Total Fertility Rate	Children Per Woman	
	POP_LIFEEXP	Life Expectancy at Birth	years	
	POP_NETMIGR	Net Migration	Per 1,000 inhabitants	
	POPDEN	Population Density	Inhabitants Per km^2	

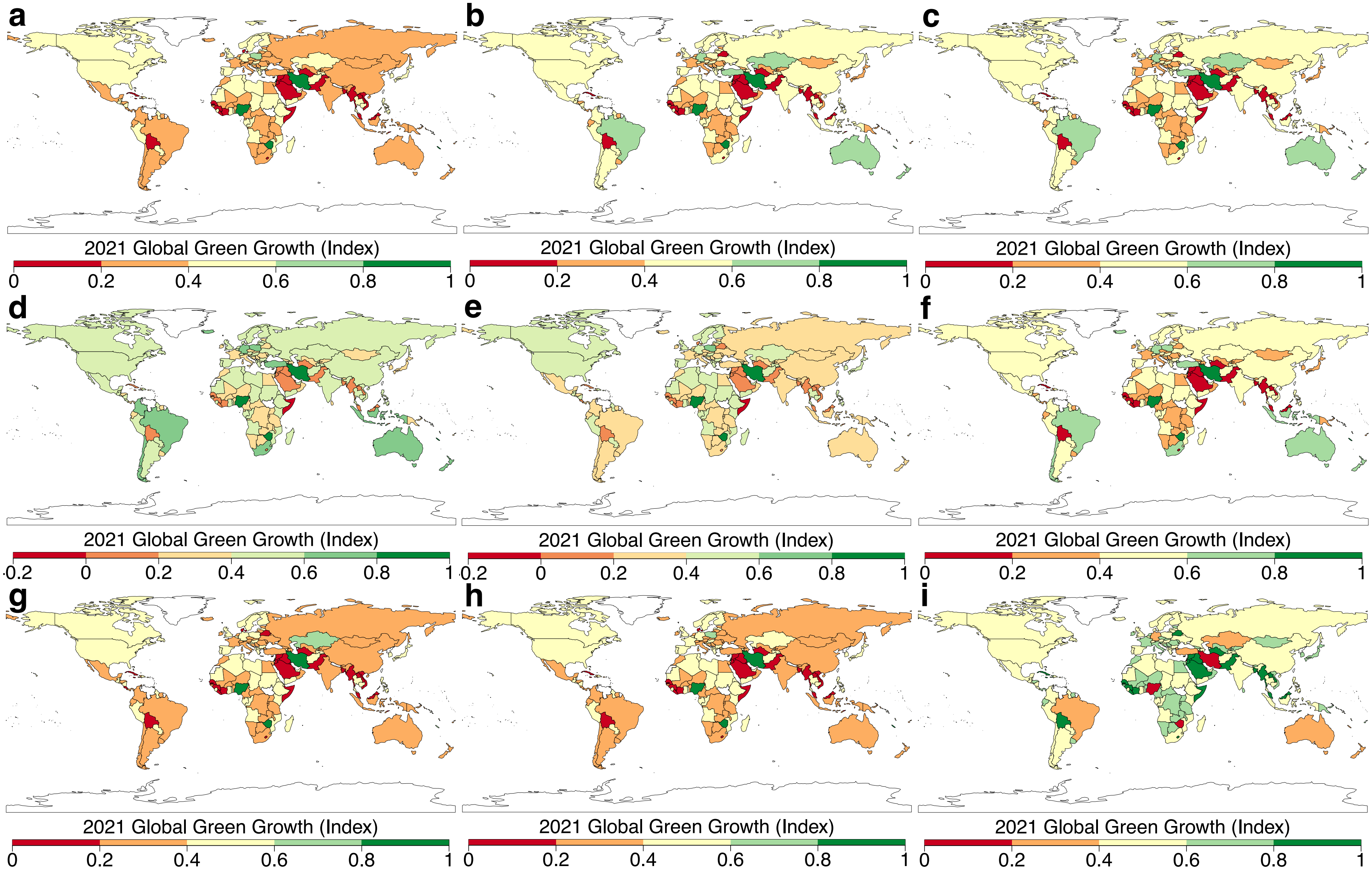
a
2021 Global Green Growth (Index)
0
0.2
0.4
0.6
0.8
1
b
2021 Global Green Growth (Index)
0
0.2
0.4
0.6
0.8
1
c
2021 Global Green Growth (Index)
0
0.2
0.4
0.6
0.8
1
d
2021 Global Green Growth (Index)
-0.2
0
0.2
0.4
0.6
0.8
1
e
2021 Global Green Growth (Index)
-0.2
0
0.2
0.4
0.6
0.8
1
f
2021 Global Green Growth (Index)
0
0.2
0.4
0.6
0.8
1
g
2021 Global Green Growth (Index)
0
0.2
0.4
0.6
0.8
1
h
2021 Global Green Growth (Index)
0
0.2
0.4
0.6
0.8
1
i
2021 Global Green Growth (Index)
0
0.2
0.4
0.6
0.8
1

Figure 1. Global distribution of green growth (Index, Reference year: 2021) (a) Model 1 (b) Model 2 (c) Model 3 (d) Model 4 (e) Model 5 (f) Model 6 (g) Model 7 (h) Model 8 (i) Model 9. Model 10 was ignored due to missing values with the function returning as an error. Legend: Model 1- Comprise socioeconomics, policy response, natural asset base, environmental quality, and environmental productivity while altering the sign (i.e., flipping implies altering the sign to move in the opposite direction) of environmental productivity, environmental quality, and natural asset base. Model 2- Comprise socioeconomics, policy response, natural asset base, environmental quality, and environmental productivity with no flipping. Model 3- Comprise socioeconomics, policy response, natural asset base, environmental quality, and environmental productivity while altering the sign of environmental productivity. Model 4- Comprise socioeconomics, policy response, natural asset base, environmental quality, and environmental productivity while altering the sign of environmental quality. Model 5- Comprise socioeconomics, policy response, natural asset base, environmental quality, and environmental productivity while altering the sign of natural asset base. Model 6- Comprise socioeconomics, policy response, natural asset base, environmental quality, and environmental productivity while altering the sign of environmental productivity and quality. Model 7- Comprise socioeconomics, policy response, natural asset base, environmental quality, and environmental productivity while altering the sign of environmental productivity, and natural asset base. Model 8- Comprise socioeconomics, policy response, natural asset base, environmental quality, and environmental productivity while altering the sign of environmental quality, and natural asset base. Model 9- Comprise socioeconomics, policy response, natural asset base, environmental quality, and environmental productivity while altering the sign of socioeconomics, policy response, natural asset base, environmental quality, and environmental productivity.
